# Circulating tumor cell and oncosome subtypes in portal and peripheral venous circulations may be used for diagnosis and prognostication of pancreatic cancer

**DOI:** 10.1038/s41698-025-01165-4

**Published:** 2025-12-05

**Authors:** Stephanie N. Shishido, Divya Suresh, Jeremy Mason, Quin Liu, Kenneth Park, Rabindra Watson, Nicholas Nissen, Srinivas Gaddam, Simon K. Lo, Stephen Pandol, Peter Kuhn

**Affiliations:** 1 Convergent Science Institute for Cancer, Michelson Center, University of Southern California, Los Angeles, CA USA; 2 Institute of Urology, Catherine & Joseph Aresty Department of Urology, Keck School of Medicine, University of Southern California, Los Angeles, CA USA; 3 Norris Comprehensive Cancer Center, Keck School of Medicine, University of Southern California, Los Angeles, CA USA; 4 Pancreatic and Biliary Diseases Program, Cedars Sinai Medical Center, Los Angeles, CA USA; 5 Department of Biomedical Engineering, Viterbi School of Engineering, University of Southern California, Los Angeles, CA USA; 6 Department of Aerospace and Mechanical Engineering, Viterbi School of Engineering, University of Southern California, Los Angeles, CA USA; 7 Department of Biological Sciences, Dornsife College of Letters, Arts, and Sciences, University of Southern California, Los Angeles, CA USA

**Keywords:** Gastrointestinal cancer, Diagnostic markers

## Abstract

Pancreatic adenocarcinoma remains one of the most devastating cancers, with limited options for early diagnosis and outcome prediction. To address these gaps, we prospectively characterized circulating tumor cells (CTCs) and oncosomes from both the peripheral (PB) and portal vein (PoVB). A total of 121 blood samples from 39 patients (75 PB, 46 PoVB) undergoing endoscopic ultrasound were profiled using multiplex immunofluorescence and computational algorithms for rare event detection. Rare event counts were significantly elevated in pancreatic cancer patients compared to normal donors (*p* = 4.71e-10). PoVB yielded greater numbers of epithelial and mesenchymal CTCs compared to PB, suggesting restricted systemic entry, while oncosomes were consistently detected in both compartments. Diagnostic accuracy was highest for PB oncosomes and PoVB CTCs. Importantly, PoVB mesenchymal CTCs correlated with 1-year survival (*p* = 0.0083), and PB oncosomes predicted poor survival (*p* = 0.0014). These findings highlight complementary diagnostic and prognostic value of PB oncosomes and PoVB CTCs in pancreatic cancer.

## Introduction

Globally, pancreatic cancer is among the most lethal malignancies, with morbidity closely mirroring mortality^1^. Early detection and diagnosis of pancreatic cancer remains a critical unmet need in oncology. Pancreatic cancer is predominantly diagnosed at an advanced stage when the tumor has already metastasized to other organs and is no longer amenable to surgical resection which provide the best treatment for long term survival^2,3^. This is primarily because early-stage pancreatic cancer is asymptomatic or with nonspecific early symptoms contributing to the fact that only 15–20% of patients diagnosed at an early stage^4^. Only limited diagnostic tests currently exist for early detection that are useful in clinical practice. Early diagnosis significantly improves survival outcomes. For patients diagnosed early, surgical intervention remains the most effective strategy for improving long-term survival. The 5-year survival rate is approximately 44% for patients diagnosed with early-stage disease, compared to only 3% for those with late-stage disease^5^.

Current diagnostic modalities for pancreatic cancer include ultrasound, multiphasic contrast-enhanced computed tomography (CT), magnetic resonance imaging (MRI) or magnetic resonance cholangiopancreatography (MRCP), positron emission tomography-CT (PET-CT) or PET-MRI, and endoscopic ultrasound (EUS). These imaging techniques are also crucial for preoperative staging, assessing resectability, evaluating treatment responses, and monitoring disease progression. For patients with uncertain diagnoses or surgical indications, endoscopic ultrasound-guided fine-needle aspiration biopsy (EUS-FNA) remains the gold standard for tumor localization and pathological confirmation^6^. EUS is a highly accurate method for the diagnosis of pancreatic inflammatory, cystic and neoplastic diseases^3,7,8^, though differentiation between benign inflammatory and malignant masses is only up to 75%^9–13^. Of importance, there is a lack of low-cost approaches to identify patients with a probable early diagnosis for a complete diagnostic evaluation using these procedures.

Liquid biopsy is an emerging technology with the potential to transform pancreatic cancer screening and early detection. Circulating tumor cells (CTCs), a key component of liquid biopsy, demonstrate high specificity for pancreatic cancer and provide a comprehensive molecular representation of the tumor. Cell-free DNA (cfDNA) analyses have shown promise for monitoring tumor burden and treatment response in pancreatic cancer^14,15^ with correlations to overall survival (OS)^16–19^, but its diagnostic utility remains limited. Advances in multi-omic approaches and improved detection technologies are particularly promising for monitoring disease progression in high-risk populations, enabling early intervention and improved outcomes^20,21^. Previous studies detecting CTCs in pancreatic cancer patients indicate a significantly shorter disease-free survival and OS times for CTC-positive patients than CTC-negative patients^21,22^. The methodology employed here was previously used to show the utility of the peripheral blood (PB) and portal vein blood (PoVB) liquid biopsy in monitoring patients with pancreatic cancer with surgically resectable disease^23^. EUS is a safe procedure with a low rate of complications, 1.1–3%^24^, that can be used for CTC sampling^25–27^.

Oncosomes are a subclass of large extracellular vesicles (LEVs), typically ranging from 1 to 10 µm in diameter, and are distinct from smaller vesicles such as exosomes and microvesicles. These vesicles are generally assembled at and shed from the plasma membrane of tumor cells and have been shown to carry a complex cargo of proteins, lipids, and tumor-derived DNA that reflect the molecular characteristics of their cells of origin. Due to their size, oncosomes are found within the cellular fraction of blood samples and are detectable using high-content imaging platforms^28^. They have been previously reported in several epithelial cancers, including prostate, colorectal, and breast cancer, and are increasingly recognized for their diagnostic and prognostic potential in cancer biology^29–32^. Their tumour-specific content, abundance in circulation, and detectability without prior enrichment make oncosomes a promising acellular biomarker for non-invasive cancer detection and monitoring.

This study provides proof of concept for a minimally invasive approach leveraging liquid biopsy technologies to enhance early detection, diagnostic workup, and outcome prediction for pancreatic cancer. We hypothesized that specific liquid biopsy analytes could provide diagnostic and prognostic information to support the clinical utility of both PB and PoVB as actionable sources for enhancing the clinical care of PDAC. The results suggest that (1) screening by measuring rare cells and oncosomes in PB can be used for early detection, and (2) measurement of CTC subtypes in EUS-guided PoVB draws can be used for prediction of clinical outcomes. This approach provides a streamlined procedure from non-invasive screening to minimally invasive diagnostic confirmation and prediction. The significance of this work lies in its potential to significantly advance pancreatic cancer care through early detection and development of management algorithms based on measuring oncosome and CTC subtypes in the peripheral and portal vein circulations.

## Results

### Patient demographics

A total of 121 samples from 39 patients undergoing EUS-FNA for the diagnostic workup of PDAC were collected for this study from different timepoints with respect to the procedure and from different anatomical locations (75 PB and 46 PoVB draws). No complication was encountered with the blood drawing. In this study, 56.4% of participants were male (*n* = 22), while 43.6% were female (*n* = 17). The median age of participants was 70 years (range: 36–90), with a median BMI of 23.7 kg/m² (range: 17.5–41.3). All enrolled patients were confirmed on histopathology to be PDAC. Three (7.7%) patients had localized disease, 26 (66.7%) patients had metastatic disease, and 10 (25.6%) patients had an unknown status of metastasis at the time of diagnosis. Patient demographics and clinical information are provided in Table 1. Follow-up time from EUS to death or last known alive date was provided for all but 2 patients, median 432.5 days (range: 1–984, average = 429.2). If follow up status was not available, the patient was excluded from survival analyses. A complete blood cell count was taken for each blood sample. The average WBC count for PDAC patient samples was 5.87 (range = 0.7–19.1; median = 5.1) million cells/mL blood.

**Table 1:** PDAC patient demographics and clinical data

Measurement	Median (range)	Measurement	*n* (%)
Age	70 (36–90)	Surgery	
BMI (kg/m^2^)	23.7 (17.5–41.3)	Yes	6 (15.4)
Size (cm)	6 (2–8)	No	32 (82.0)
Gender	*n* (%)	N/A	1 (2.6)
Male	22 (56.4)	Metastatic disease	
Female	17 (43.6)	None	3 (7.7)
Race		N/A	11 (28.2)
Asian	4 (10.3)	Ascites	1 (2.6)
Black	5 (12.8)	Bone	1 (2.6)
White	25 (64.1)	Breast	1 (2.6)
Other	4 (10.3)	Kidney	1 (2.6)
NA	1 (2.6)	Liver	12 (30.8)
Ethnicity		LN	3 (7.7)
Hispanic	6 (15.4)	Lung	5 (12.8)
Non-Hispanic	32 (82.1)	Peritoneal	4 (10.3)
N/A	1 (2.6)	Suspicious for liver	2 (5.1)
KRAS mutation		Disease response	
KRAS G12C	1 (2.6)	Stable	5 (12.8)
KRAS G12D	7 (17.9)	Progressive	25 (64.1)
KRAS G12R	3 (7.7)	Recurrent	1 (2.6)
KRAS G12V	6 (15.4)	N/A	8 (20.5)
None	21 (53.8)		
N/A	1 (2.6)		

“Suspicious for liver” indicates a clinical observation of a potential liver lesion but not confirmed as a metastatic site. “None” indicates no evidence of metastasis. “NA” denotes unavailable clinical data.

### Rare event identification in PDAC diagnostic workup and NDs

**Fig. 1:**
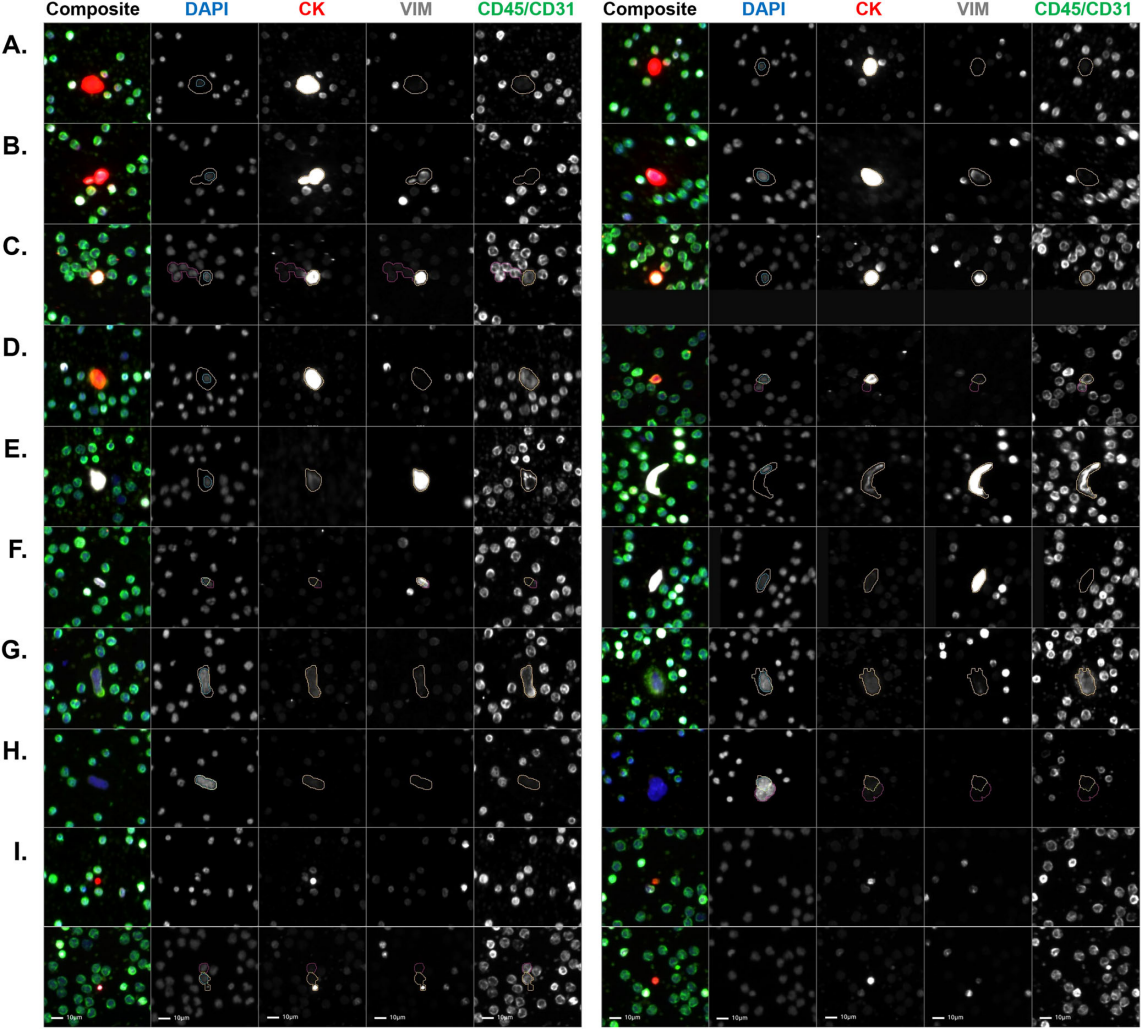
Representative gallery of rare events detected in the liquid biopsy of PDAC patients during EUS procedure. The composite image and each individual biomarker channels are provided. **A** Epi.CTC; **B** Mes.CTC; **C** D | CK | V | CD, **D** D | CK | CD, **E** D | V | CD, **F** D | V, **G** D | CD, **H** DAPI-only, **I** oncosomes (top left: CK onc, top right: CK | CD onc, bottom left: CK | V onc, bottom right: CK | V | CD onc examples). Images taken at ×100 magnification. Scale bar = 10 µm. Blue: DAPI, Red: CK, White: VIM, Green: CD45/CD31.

A representative gallery of rare events (8 cellular and 4 oncosome categories) with variable expression profiles detected in the PDAC cohort are shown in Fig. 1. Oncosomes were identified by a lack of nuclear structure, round morphology, positive CK signal, and variable expression of Vim and CD.

**Fig. 2:**
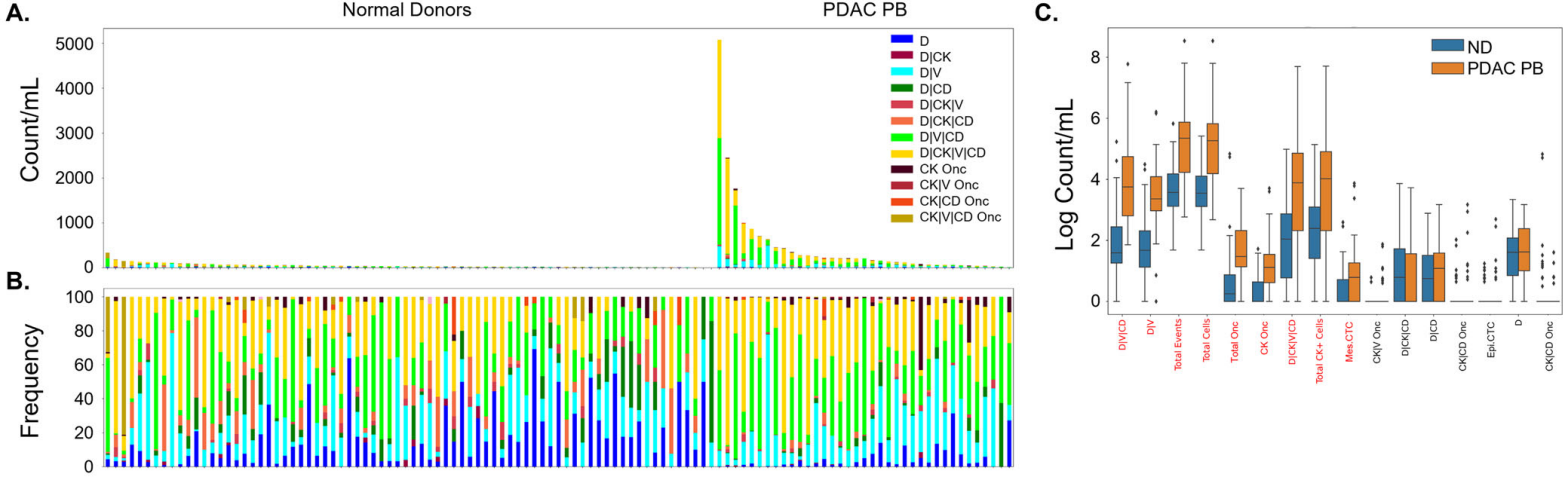
Rare event detection in PB samples collected before EUS of PDAC patients compared to NDs using HDSCA3.0. **A** Enumeration and **B** frequency of each rare event by channel-type classification. **C** Logarithmic box and whisker plots of the channel-type rare events/mL, with the center line representing the median, box limits showing upper and lower quartiles, and whiskers representing 1.5× interquartile range. The points represent outliers. The liquid biopsy analytes in red text are the statistically significant variables detected between PDAC and ND samples, sorted from lowest (left) to highest (right) *P*-value.

PB samples taken prior to EUS procedure were compared to ND control samples to determine the presence of cancer-related rare events in circulation. PDAC PB draws had a significantly greater incidence of total rare events (cellular and oncosomes), total cells, total oncosomes, total CK expressing cells, and specific cellular phenotypes (D | V | CD, D | V, D | CK | V | CD, Mes.CTC) and CK+ oncosomes (Fig. 2). This indicates that there are unique biomarkers of disease present in the PB pre-EUS draw of PDAC patients. PDAC PB and ND analyte incidence is provided in Supplementary Table 1 and significant differences between comparisons are provided in Supplementary Table 2.

Further analysis was conducted to determine if the EUS procedure affects the circulating analytes in PDAC patients, we compared the PB analytes before and after EUS-FNA. At the cohort level analysis, there was a single cellular phenotype that was identified to be present at a higher incidence pre-procedure than post-procedure (D | CK | V | CD, *P*-value = 0.0495; Supplementary Fig. 1). In a paired analysis of time points per patient the pre-procedure had a significantly higher incidence of total events (*P*-value = 0.0209), total cells (*P*-value = 0.0177), total CK expressing cells (*P*-value = 0.0041), and D | CK | V | CD (*P*-value = 0.0039). All other PB analytes were not significantly different between timepoints. This indicates that the EUS procedure does not lead to additional shedding of rare analytes into the circulation.

### Rare event identification in PoVB and PB of EUS diagnostic workup patients

**Fig. 3:**
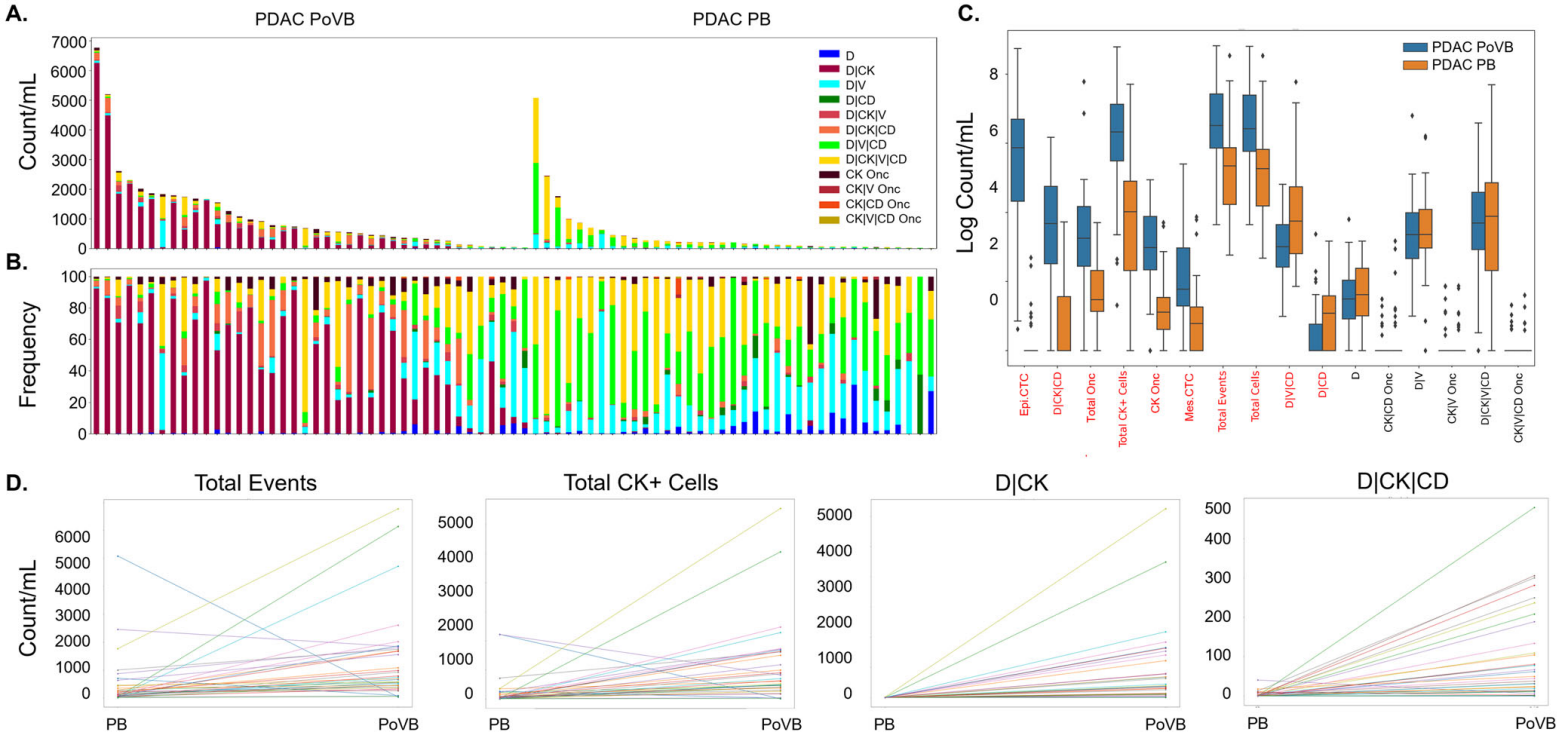
Rare event detection in PB and PoVB samples collected from PDAC patients using HDSCA3.0. **A** Enumeration and **B** frequency of each rare event by channel-type classification. **C** Logarithmic box and whisker plots of the channel-type rare events/mL, with the center line representing the median, box limits showing upper and lower quartiles, and whiskers representing 1.5× interquartile range. The points represent outlines. The red text represents the statistically significant variables between PB and PoVB samples, sorted from lowest (left) to highest (right) *P*-value. **D** Matched analysis of the most significant differences in channel-type rare events/mL between anatomical locations.

To determine if the liquid biopsy collected from the draining vein downstream of the pancreas (i.e., PoVB) is more enriched for tumor associated analytes, the PoVB collected during EUS was compared to the pre-procedure PB of PDAC patients (Fig. 3). Compared to pre-procedure PB, the PoVB draws had a higher incidence of total rare events, total rare cells, total CK expressing cells, total oncosomes and multiple phenotypic specific cellular categories (Epi.CTC, D | CK | CD, Mes.CTC, D | V | CD, and D | CD) and CK oncosomes. The most significant differences were observed in the Epi.CTC and D | CK | CD cellular classifications (*p*-value < 1E-06 matched analyses). All other PB analytes were not significantly different. This indicates that the two anatomical locations differ significantly, highlight a compartment-specific distribution of circulating analytes, with CTCs significantly enriched in PoVB and largely absent in PB, whereas oncosomes were detectable in both compartments, making them a viable clinical marker.

To contextualize the findings across disease stages, we included a supplemental analysis (Supplemental Table 3) comparing analyte profiles between localized and metastatic PDAC cases within this EUS cohort, as well as in our previously published cohort of surgically resectable patients^23^. This analysis supports the presence of rare circulating analytes even in early-stage disease.

### Rare cell characterization

The heterogeneity of the rare cell population detected in the PB and PoVB samples was explored through cellular morphometrics. Detailed phenotypic characterization of rare cell morphometrics is presented in Supplementary Figs. 2 and 3, supporting the observed compartmental differences and phenotypic heterogeneity.

#### Patient level prediction of cancer

With the goal of determining if the PB or PoVB could be used to detect cancer in an individual, we first utilized a logistic regression for univariate analysis using the enumerations of total or individual rare events from ND and PDAC patient samples. Table 2 shows the percent accuracy of predicting cancer from non-cancer using each category of analyte. Total events were the strongest predictor for correctly classifying into cancer and normal for both PoVB and PB. In PoVB, the total CK+ cell population (i.e., CTCS) was also a strong predictor, while in the PB the total cell population was a strong predictor, this trend with the observation that PoVB is enriched for CK+ cells. The CK oncosomes were the next strongest predictor for both anatomical locations. The PoVB predictions were driven by Epi.CTCs. The PB predictions were driven by D | V | CD phenotypic cells, which are morphologically consistent with endothelial cells.

**Table 2:** Univariate logistic regression analysis for predicting cancer vs. non-cancer

PB		PoVB	
Rare event	Accuracy (%)	Rare event	Accuracy (%)
CK | V | CD Onco	65.22	CK | CD Onco	58.33
D	65.22	CK | V | CD Onco	62.50
D | CK | CD	65.22	D	62.50
CK | CD Onco	65.22	D | CD	62.50
D | CD	65.22	D | V | CD	66.67
Total Oncosomes	65.22	CK | V Onco	70.83
Epi.CTC	65.22	D | CK | CD	83.33
D | V	69.57	D | V	83.33
Mes.CTC	69.57	Total Oncosomes	83.33
CK | V Onco	73.91	D | CK | V | CD	87.50
Total CK+ Cells	73.91	Mes.CTC	87.50
D | CK | V | CD	78.26	Epi.CTC	91.67
D | V | CD	86.96	Total Cells	95.83
CK Onco	86.96	CK Onco	95.83
Total Cells	91.30	Total CK+ Cells	95.83
Total Events	91.30	Total Events	95.83

Next, we tested a multivariate analysis via six different models for both PB and PoVB to associate with the presence of cancer versus no cancer (Table 3). The most important features in PB associated with the presence of cancer were oncosomes determined by logistic regression with an accuracy of 95.65%. The most important marker for prediction of clinical outcomes were Epi.CTCs with an accuracy of 100% in PoVB. These findings highlight the potential of distinct circulating tumor-related analytes as robust predictors, varying by blood compartment, and underscore the utility of tailored modeling approaches for optimized accuracy.

**Table 3:** Multivariate analysis for predicting cancer vs. non-cancer by anatomical draw

Model	PB accuracy (%)	PoVB accuracy (%)
Logistic regression	95.65	95.83
Decision tree	82.61	100.00
Random forest	82.61	95.83
SVM	78.26	95.83
Gradient boosting	78.26	100.00
Neural network	82.61	45.83

### Correlations with clinical data

To explore the potential clinical significance between the liquid biopsy analytes detected in PDAC patient samples collected at EUS, correlation analyses were conducted between PB and PoVB rare event occurrences and clinical data elements. All significant correlations are provided in Table 4. There were no significant correlations identified for KRAS mutation, ethnicity, and race. Survival status, survival time from EUS, and patient age were the most common variables to correlate with the liquid biopsy analytes. The PB was predictive of survival status, gender, and mass location in the body of the pancreas. The survival time from EUS and patient age correlated across anatomical locations. The PoVB was associated with the location of the mass, specifically the mass location being in the tail or head of the pancreas, suggesting the liquid biopsy can provide tumor specific information.

**Table 4:** Significant correlations between the clinical/demographic variables and the rare events identified by HDSCA3.0 in the liquid biopsy samples collected from the PoVB and PB (Pre-procedure) from PDAC patients

Variable	Rare event	Clinical metric	Correlation method	Correlation value	*P*-Value
PB	CK | V Onco	Age	Spearman	0.3834	0.0191
PB	CK Onco	Gender	Wilcoxon Rank Sum	−2.4066	0.0161
PB	D | CD	Mass Location: body	Wilcoxon Rank Sum	−2.1594	0.0308
PB	D | V | CD	Survival Status	Wilcoxon Rank Sum	−3.2609	0.0011
PB	D | CK | V | CD	Survival Status	Wilcoxon Rank Sum	−2.3314	0.0197
PB	Total CK+ Cells	Survival Status	Wilcoxon Rank Sum	−2.3162	0.0205
PB	Total Cells	Survival Status	Wilcoxon Rank Sum	−2.2552	0.0241
PB	Total Events	Survival Status	Wilcoxon Rank Sum	−2.2552	0.0241
PB	D | V | CD	Survival Time from EUS	Spearman	−0.3866	0.0218
PB	CK | CD Onco	Survival Time from EUS	Spearman	−0.3717	0.0279
PB	D | CK | CD	Survival Time from EUS	Spearman	−0.3518	0.0382
PoVB	Mes.CTC	1-year Survival	Wilcoxon Rank Sum	2.6404	0.0083
PoVB	Total Oncosomes	Age	Spearman	−0.3771	0.0214
PoVB	Total Events	Age	Spearman	−0.3440	0.0371
PoVB	Total Cells	Age	Spearman	−0.3351	0.0426
PoVB	Total CK+ Cells	Age	Spearman	−0.3326	0.0443
PoVB	D | V	Location of Mass	Wilcoxon Rank Sum	2.1213	0.0339
PoVB	D | CK | CD	Mass Location: head	Wilcoxon Rank Sum	2.6819	0.0073
PoVB	D | CK | CD	Mass Location: Tail	Wilcoxon Rank Sum	−2.7390	0.0062
PoVB	D	Survival Time from EUS	Spearman	−0.4475	0.0070
PoVB	Mes.CTC	Survival Time from EUS	Spearman	−0.3628	0.0322
PoVB	D | V	Survival Time from EUS	Spearman	−0.3530	0.0375

The PoVB was correlated with 1-year survival in which specifically there was a positive correlation with Mes.CTCs (*P*-value = 0.0083). This suggests that the Mes.CTC count detected in the PoVB is a predictor of patient outcome. Additionally, the total CK expressing rare cell population (total CTC population) correlated with survival status (pre-procedure PB), indicating that the CTCs detected in the PB pre-procedure can be predictive of patient outcome.

**Fig. 4:**
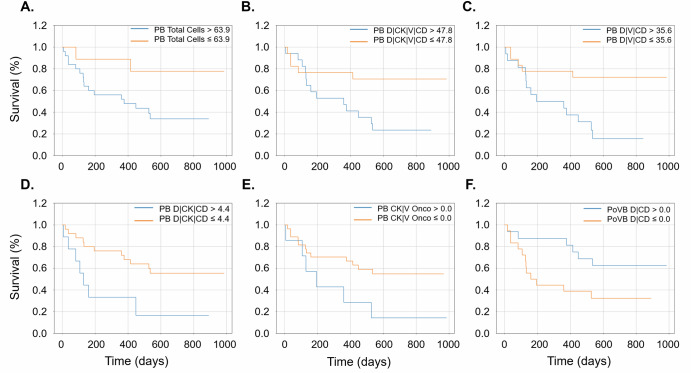
Overall survival analysis of rare events in PB and PoVB samples taken during diagnostic workup. Kaplan–Meier (KM) curves showing the patients stratified by **A** PB total cells, **B** PB D | CK | V | CD, **C** PB D | V | CD, **D** PB D | CK | CD, **E** PB CK | V Oncos, **F** PoVB D | CD. A complete list of all significant analytes and thresholds related to overall survival are provided in Supplementary Table 3.

Next, an analysis of circulating rare events identified in PB and PoVB samples correlated to pancreatic cancer patient outcome revealed that total events, total cells, total CK+ cells, and specific phenotypes of cells (D | CK | V | CD, D | CK | CD, D | V | CD, DAPI only) and CK | V Oncos stratify patients for OS with values greater than the threshold indicative of poor outcome (Fig. 4; and Supplementary Table 4). Interestingly, the D | CD phenotype was the only rare event type that was an indicator of patient survival when above the threshold value (0 cells/mL). The majority of the rare events indicative of patient outcome were detectable in the PB. Together, these findings indicate that PB samples collected during diagnostic workup can serve as a valuable predictor of patient outcomes in pancreatic cancer, with specific circulating rare event phenotypes strongly correlating with OS.

## Discussion

This proof of concept study demonstrates the ability of an enrichment free liquid biopsy approach to detect cancer associated analytes unique to patients undergoing the diagnostic workup for pancreatic cancer via the EUS procedure. Importantly, we show the safety and feasibility of a PoVB draw during EUS, which provided an enrichment of CTCs and correlation with patient outcome. While multiple rare event types were measured, our findings indicate that oncosomes in PB and mesenchymal CTCs in PoVB are the most robust and clinically informative markers in this cohort. This suggests liquid biopsy can serve as a companion diagnostic tool in pancreatic cancer, with PB utilized during screening and EUS-guided PoVB draws allowing for development of prediction modeling.

Screening for pancreatic cancer through PB holds significant promise, particularly given the dramatic improvement in survival rates when the disease is detected early and treated surgically. Previous studies have shown the capability of detecting circulating analytes in PB and PoVB from surgically resectable, early-stage PDAC patients^23,33,34^. This EUS study reinforce the potential of PB sampling to detect and classify circulating rare cells and oncosomes, highlighting a non-invasive approach to cancer screening. By leveraging advanced analytical techniques, we further demonstrate the cellular heterogeneity within PB, revealing critical distinctions in both total counts and specific phenotypes of circulating analytes between pancreatic cancer patients and ND controls. These statistically significant differences underline the sensitivity and specificity of PB analysis in distinguishing individuals with PDAC from healthy individuals. The findings presented here emphasize the potential of a simple PB draw as a valuable screening tool, enabling earlier detection and improved outcomes for individuals at risk of pancreatic cancer. Although the majority of patients in this cohort presented with advanced disease, this reflects the current diagnostic landscape of pancreatic cancer, which is typically identified at a late stage. As such, our findings are representative of diagnostic utility during clinical evaluation rather than population-level early detection. We further acknowledge that the healthy donor controls were not age- or procedure-matched. To address this limitation, future analyses will include samples from patients with benign pancreatic conditions, which are currently being accrued, to more rigorously assess the specificity of circulating analytes for pancreatic cancer when compared to benign pancreatic diseases.

PoVB sampling offers a transformative approach to enhance the definitive diagnosis of pancreatic cancer and provides an opportunity to develop prediction models for clinical outcomes. This study emphasizes the importance of understanding the logistics of PoVB collection during EUS procedures, which were demonstrated to be safe, without promoting the dissemination of CTCs, as confirmed by matched pre- and post-procedure analysis of PB samples. PoVB was found to be enriched with cancer-associated analytes, particularly CTCs, compared to PB, underscoring the advantage of sampling closer to the tumor’s anatomical location^23,26,33–35^. This proximity results in markedly increased sensitivity for detecting tumor-related biomarkers. Moreover, the CTCs isolated from PoVB samples can undergo detailed molecular characterization, providing critical insights for a definitive diagnosis and prognosis^25^. Future studies will build on these findings, employing advanced single-cell genomics and targeted proteomics to further unravel the molecular underpinnings of pancreatic cancer, with the potential to refine diagnostic precision and guide personalized treatment strategies.

The analysis of circulating rare events in pancreatic cancer highlights distinct roles for different sample types and event subtypes in detection and outcome prediction. PB appears most effective for detecting cancer, with circulating endothelial cells being the most abundant analyte detected and serving as strong indicators of diagnosis (Table 2) and predictors of survival (Fig. 4). Furthermore, CK-positive oncosomes in PB are particularly promising for diagnostic purposes, distinguishing non-diseased individuals from pancreatic cancer patients (Table 2), while CK | V-positive oncosomes in PB correlate with survival outcomes (Fig. 4). These findings underscore the utility of PB samples, especially for early cancer detection and prognostic assessments, with oncosome subtypes demonstrating specificity for various clinical parameters, including age, gender, and survival time from EUS (Table 4). In contrast, PoVB samples excel in predicting patient outcomes, particularly through the detection of Mes.CTCs. While Epi.CTCs in PoVB show no significant correlation with clinical outcomes, Mes.CTCs demonstrate a strong association with 1-year survival (Table 4), highlighting their prognostic value. PoVB samples also provide a broader diagnostic utility, encompassing the CK-positive cellular populations, as well as CK-positive oncosomes (Table 3). These observations suggest that PoVB samples may capture a more diverse array of tumor-related analytes critical for understanding disease progression and patient prognosis. Taken together, these findings emphasize the complementary roles of PB and PoVB in liquid biopsy applications. PB excels in cancer detection and survival prediction through endothelial cells and oncosomes, while PoVB is better suited for predicting outcomes via Mes.CTCs. These insights demonstrate the importance of both sample types and event subtypes in improving diagnostic and prognostic approaches in pancreatic cancer.

The comparison between PoVB and PB reveals that CTCs are actively released from the pancreas into the portal vein but rarely enter systemic circulation, as indicated by their markedly lower incidence in PB. This disparity suggests that the liver may act as a biological filter, capturing CTCs and potentially explaining the high frequency of liver metastases observed in pancreatic cancer. Interestingly, oncosomes that carry a complex cargo of metabolites, proteins, and genetic material are not similarly filtered and are readily detected in PB. These oncosomes, which have been implicated in other epithelial cancers such as bladder, prostate, breast, and colorectal cancers, underscore their clinical and biological relevance as a unique class of analytes. Their presence in PB highlights their potential as systemic biomarkers for cancer detection and progression monitoring. Future studies will focus on the detailed characterization of oncosomes, seeking to confirm their association with cancer progression and to elucidate their specific roles in the biology and clinical trajectory of PANC.

The data presented in this study suggest that CTCs released into the circulation from the PoVB may be rapidly cleared, potentially disappearing in a single circulatory cycle. The portal vein supplies up to 1 l of blood per minute to the liver (600–900 mL/min^36,37^), translating to a staggering volume of blood and cells moving through this system daily. Here we identified an average CTC count (estimated by total CK expressing cell) of 560/mL, which at a flow rate of 1 L/min over 24 h, that’s about 800 million CTCs passing through the portal circulation every day. Despite this high cellular turnover there is a markedly reduced load of CTCs which we observed in the PB. Given the liver receives 75% of its blood supply from the portal vein and the hepatic vein accounts for 25% of total blood outflow^38^, it is estimated that approximately 19% of the blood in circulation is supplied directly from the portal vein. This raises intriguing questions: where do these cells go? Are they being captured and metabolized by the liver, cleared by immune surveillance mechanisms, or sequestered elsewhere within the body? Understanding the mechanisms of cell clearance and distribution from PoVB to PB is critical for developing more accurate liquid biopsy approaches and could uncover novel biological processes that govern tumor cell survival and dissemination.

An increased number of CTCs is strongly associated with worse survival outcomes, particularly within the first year of diagnosis or treatment. In this study, we demonstrate that both the quantity and specific subtypes of CTCs identified in liquid biopsy samples are predictive of clinical states and outcomes. This highlights the diagnostic and prognostic value of CTCs as biomarkers, offering a minimally invasive method to stratify patients based on their likely progression. While other clinical and molecular factors undoubtedly contribute to patient outcomes, our findings underscore the central role of CTC dynamics in shaping survival trajectories. We acknowledge that our survival analyses may be underpowered due to the limited cohort size and the small number of early-stage patients. This study was designed as a preliminary, proof-of-concept investigation to explore the prognostic relevance of circulating analytes detected during the diagnostic workup of PDAC. While some findings reached statistical significance, including the association between PoVB mesenchymal CTCs and 1-year survival, others showed strong trends that may not have achieved significance due to sample size constraints. Importantly, the magnitude of observed effect sizes suggests biologically meaningful differences that merit further investigation in larger, independent cohorts. These preliminary signals pave the way for future investigations with larger sample sizes and longer follow-up using multivariate analyses to integrate CTC data with other clinical and molecular predictors, creating a more comprehensive framework for predicting patient outcomes. This approach holds promise for tailoring treatment strategies, enhancing personalized medicine, and improving OS in cancer patients.

To differentiate our study from other PoVB studies, it is essential to highlight key methodological and performance distinctions. One notable contrast is with CellSearch^26,27,35^, which relies on capturing CTCs based on their expression of CK and epithelial cell adhesion molecule (EpCAM). This approach is inherently limited to CTCs exhibiting epithelial markers, thereby excluding mesenchymal or otherwise atypical CTCs, which are critical in the context of tumor heterogeneity and progression^25^. Our methodology, however, addresses these limitations by employing a broader, more sensitive approach enabling the detection of a wider array of tumor-related analytes. In PB, where CTCs are often present at extremely low levels or even absent, the sensitivity of our technology ensures that critical tumor signals are still detectable. This capability markedly enhances the clinical utility of our approach in comparison to CellSearch, particularly in cases where traditional methods would yield no significant findings.

Circulatory data offers critical insights into the dynamic behavior of malignancies and their interactions with host physiology. Tumor cell dwell time within the bloodstream reflects the malignancy’s aggressiveness, with rapid circulation and substantial outflow from the PoVB suggestive of advanced disease and increased metastatic potential. The relationship between metastases and the liver’s metabolic state underscores the importance of liver health in influencing outcomes. Conditions such as diabetes or preexisting liver disease may alter liver function, impacting its capacity to filter tumor cells or metabolize therapeutic agents. Understanding these factors could provide novel insights into pancreatic cancer biology. Future studies should investigate how liver health, assessed through metabolic and disease-specific markers, affects pancreatic cancer progression and metastasis. Multivariate predictive models that incorporate liver function metrics alongside CTC characteristics could refine outcome predictions and guide therapeutic interventions. Similarly, the incorporation of CA19-9 measurements, radiographic staging, and EUS findings can enable comparisons to current diagnostic standards and integrative modeling. Such efforts will be critical to determine the additive or complementary value of liquid biopsy to existing clinical workflows, particularly in differentiating malignant from benign lesions during diagnostic workup. By integrating these parameters, we can develop a more nuanced approach to understanding cancer progression and tailoring treatment strategies, ultimately improving patient care.

## Methods

### Study design

A total of 39 patients were recruited at Cedars-Sinai Medical Center, undergoing EUS-FNA for diagnosis and staging of pancreatic cancer (PDAC). PB samples were taken immediately before and after the procedure, and PoVB samples were collected during the procedure prior to manipulation of the pancreas. Samples were collected between August 2021 to October 2023. The study was conducted according to the guidelines of the Declaration of Helsinki and approved by the Institutional Review Board (or Ethics Committee). Patient recruitment was conducted in accordance with the Institutional Review Board protocols, and all patients provided written informed consent. All enrolled participants completed the study, there was no attrition. PB samples from 76 normal donors (ND) with no known pathology were procured from Epic Sciences and Scripps Normal Blood Donor Service for comparison. Randomization was not applicable to this observational proof-of-concept study, and no power analysis was conducted. All analyses were performed in a blinded manner, ensuring that the liquid biopsy assessments were conducted without knowledge of sample type or cohort.

### Patient and public involvement

Patients or the public were not involved in the design, or conduct, or reporting, or dissemination plans of our research.

### Portal venous blood sampling

PoVB collection under EUS guidance was first reported on humans in 2015, using a 19 g FNA needle on 18 patients with pancreaticobiliary cancers^27^. Limited by needle stiffness and perceived bleeding risk of the 19 g needle, we assessed the possibility of using a softer and thinner 22 G FNA needle for that purpose. After confirming feasibility and safety of a 22 g needle in a swine model^39^, we designed this study with obtaining PoVB from patients with newly-discovered presumed pancreatic cancer undergoing EUS evaluations at Cedars-Sinai Medical Center. Each consented patient was sedated with propofol anesthesia. A PB sample was drawn from an arm vein prior to the EUS procedure, along with aspiration of main PoVB with a 22 g EUS-FNA needle during procedure. All blood samples (8 mL) were collected in 10 mL blood collection tubes (Cell-free DNA, Streck, La Vista, NE, USA). There were no adverse events related to PoVB sample collection.

### Blood sample processing

PB and PoVB samples were processed as previously described^40–42^ within 48 h of collection, with comparable timing across patient and ND samples. Briefly, red blood cells underwent lysis with an isotonic ammonium chloride solution and the nucleated cell fraction was plated at approximately 3 million cells per custom glass slide (Marienfeld, Lauda, Baden-Württemberg, Germany) prior to 7% BSA clocking and long-term cryostorage. Patient 9 samples were not included due to low cellularity.

### Blood sample staining and imaging

Two slides per sample test were in the IntelliPATH FLXTM autostainer (Biocare Medical LLC, Irvine, CA, USA) as previously described^31,43–46^. Samples were fixed with 2% paraformaldehyde and stained with anti-human CD31: Alexa Fluor 647 mAb (clone: WM59, MCA1738A647, BioRad, Hercules, California, USA; RRID:AB_322463), anti-mouse IgG monoclonal Fab fragments (115-007-003, Jackson ImmunoResearch, West Grove, Philadelphia, USA), and permeabilized with 100% cold methanol. This was followed by two additional incubations: (1) a antibody cocktail including: anti-human vimentin (Vim; clone: D21H3, 9854BC, Cell Signaling, Danvers, MA, USA; RRID:AB_1082935), anti-human cytokeratins (CKs) 1, 4, 5, 6, 8, 10, 13, 18, and 19 (clones: C-11, PCK-26, CY-90, KS-1A3, M20, A53-B/A2, C2562, Sigma, St. Louis, MO, USA; RRID:AB_476839), anti-human CD45:Alexa Fluor® 647 (clone: F10-89-4, MCA87A647, AbD Serotec, Raleigh, NC, USA; RRID:AB_324730), and anti-human CK 19 (clone: RCK108, GA61561-2, Dako, Carpinteria, CA, USA), and (2) Alexa Fluor 555 anti-mouse IgG1 antibody (A21127, Invitrogen, Carlsbad, CA, USA; RRID:AB_141596) and 4′,6-diamidino-2-phenylindole (DAPI; D1306, ThermoFisher, Waltham, MA, USA; RRID:AB_2629482). Finally, slides were mounted using a glycerol-based aqueous mounting media and imaged using automated fluorescence scanning microscopy at ×100 magnification across the four channels (DAPI, AlexaFluor® 488, AlexaFluor® 555, AlexaFluor® 647) for 2304 frames per slide.

### Rare event detection and classification

Rare event identification utilized the computational algorithm Outlier Clustering Unsupervised Learning Automated Report (OCULAR)^23,31,43,44,46^. The candidate rare events were further subjected to a manual data curation process by multiple trained technicians to verify signal intensity and distribution, as well as morphology. To ensure consistency and accuracy, all algorithm-generated candidate events were reviewed by at least two independent trained analysts using a standardized review pipeline. Analysts were blinded to sample identity and trained to consensus using a validated set of reference images. Discrepancies were adjudicated jointly, and only consensus-classified events were included in the final dataset. Prior work from our group has demonstrated high inter-observer agreement for rare event classification under this protocol. In addition, replicate slides from the same patient sample routinely demonstrate consistent classification frequencies and morphometric distributions, supporting both analytical and technical reproducibility of the platform.

Rare events detected were classified into eight cellular and four oncosome categories based on the presence of fluorescent signal in each channel as mentioned previously^23,31,43,44,46^. These categories represent a simplified phenotypic framework applied to a biologically complex set of circulating analytes. Epithelial-like CTCs (epi.CTCs) were defined as rare cells positive for CK, and negative for VIM and CD45/CD31 (D | CK). Mesenchymal-like CTCs (mes.CTC) were classified as rare cells with positive CK and VIM signal, and CD45/CD31 negative (D | CK | V). Additional CTC categories of interest include rare cells with CK and CD45/CD31 signal (D | CK | CD), which may represent immune-associated^47^ or platelet-coated tumor cells^43^, and triple positive cells with signal in all channels (D | CK | V | CD), which could include mesenchymal-like cells^44^. These categories highlighting biologically distinct populations despite overlapping markers. Other interesting cell categories include: VIM positive (D | V), VIM and CD45/CD31 positive (D | V | CD), CD45/CD31 positive (D | CD), and cells with only morphologically distinct nuclei (DAPI only). White blood cells (WBC) in whole blood were automatically quantified (Medonic M-series Hematology Analyzer, Clinical Diagnostic Solutions Inc., Fort Lauderdale, FL, USA) and used to present rare event enumerations as events/mL. Given the inherent variability and overlapping features of rare circulating cells, analyses were performed both at the individual subtype level and as aggregate categories (e.g., total oncosomes, total CK-expressing cells, total rare cells, total events) to reduce overfitting and better assess clinical associations.

### Statistical analysis

To determine the correlations between time and location parameters and rare events detected in the liquid biopsy samples, non-parametric statistical tests were used, with p-values below 0.05 considered as statistically significant. Mann–Whitney U or Wilcoxon rank-sum test was applied to compare NDs with PDAC patients (Python package scipy version 1.10.1; RRID:SCR_008058). For paired samples, matched by locations and draw times, the Wilcoxon signed-rank test was used. Cellular morphometrics were used to investigate the heterogeneity of the rare-event population. Cell heterogeneity was visualized via morphometric probability distributions and two-dimensional tSNE (t-distributed stochastic neighbor embedding) plots. Clinical correlations between PDAC pre-procedure PB and PoVB rare event occurrence and clinical data elements were analyzed using the Wilcoxon rank sum test for categorical variables and Spearman’s rank correlation for continuous and ordinal variables. All code developed is standard and easily reproducible.

### Ethics statement

This study involves human participants and was approved by the following Institutional Boards: the Cedars-Sinai Medical Center, Office of Research Compliance and Quality Improvement, Protocol IRB No. Pro00041571, study initiation approved 9 November 2020, continuing review approved on 16 August 2023; and the University of Southern California Institutional Review Board, Protocol No. HS-20-00974, approved on 2 February 2021 for coded specimens/data.

## Supplementary information

Supplementary Material

## Data Availability

All data discussed in this manuscript are included in the main manuscript text or supplementary materials. The imaging data are available through the BloodPAC Data Commons, Accession ID “BPDC000154” https://data.bloodpac.org/open-BPDC000154. To be provided prior to publication.

## Code availability

All code developed is standard and easily reproducible.
