# Supplementary material for: Circulating tumor cell and oncosome subtypes in portal and peripheral venous circulations may be used for diagnosis and prognostication of pancreatic cancer
Source: NPJ Precis Oncol. 2025 Dec 5;9:397. doi: 10.1038/s41698-025-01165-4 (PMC12706006; doi:10.1038/s41698-025-01165-4)
Supplement: Supplementary file 1 — Supplementary Material [file 41698_2025_1165_MOESM1_ESM.pdf]

Supplemental Material

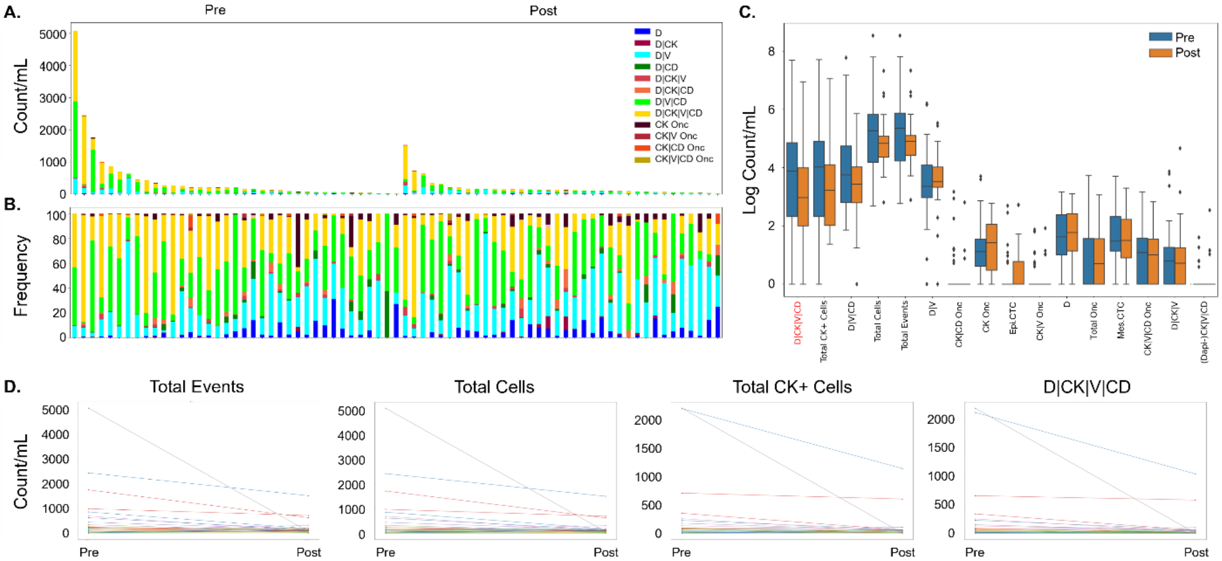

Supplemental Figure 1. Rare event detection in PB samples collected before and after EUS procedure of PDAC patients using HDSCA3.0. A) Enumeration and B) frequency of each rare event by channel-type classification. C) Logarithmic box and whisker plots of the channel-type rare events/mL, with the center line representing the median, box limits showing upper and lower quartiles, and whiskers representing 1.5x interquartile range. The points represent outlines. The red text represents the statistically significant variables between pre- and post-procedure samples. D) Matched analysis of the significant differences in channel-type rare events/mL between timepoints.

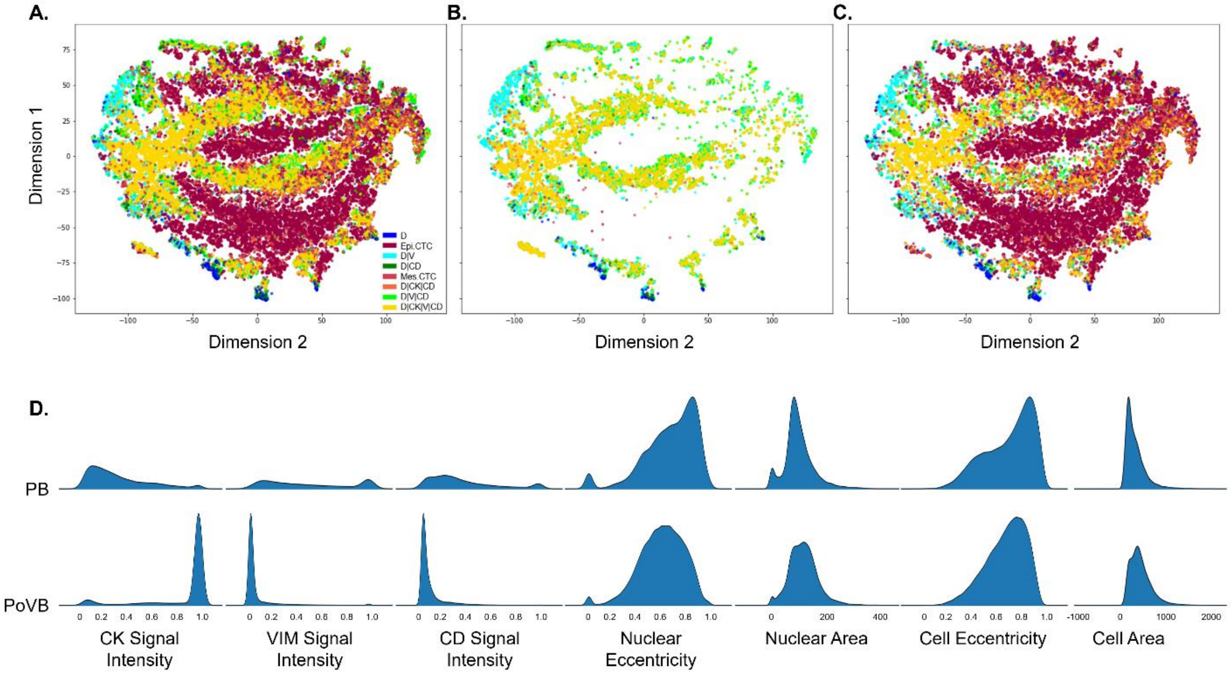

Supplemental Figure 2. Graphical representation of the different channel-type rare cells/mL between anatomical locations. Morphology tSNE plot of rare cells by channel-type classification in A) and separated by B) PB and C) PoVB. D) Probability density distribution plots for select morphometric parameters across channel-type classifications of median CK signal intensity, median VIM signal intensity, median CD45/CD31 signal intensity, nuclear eccentricity, nuclear area, cellular eccentricity, and cellular area. The Y-axis remains consistent across all

conditions, while X-axis varies depending on the specific parameters. The area under a density curve is always equal to 1. This is because a density curve represents a probability distribution, and the total probability of all possible outcomes within a distribution must sum to 1.

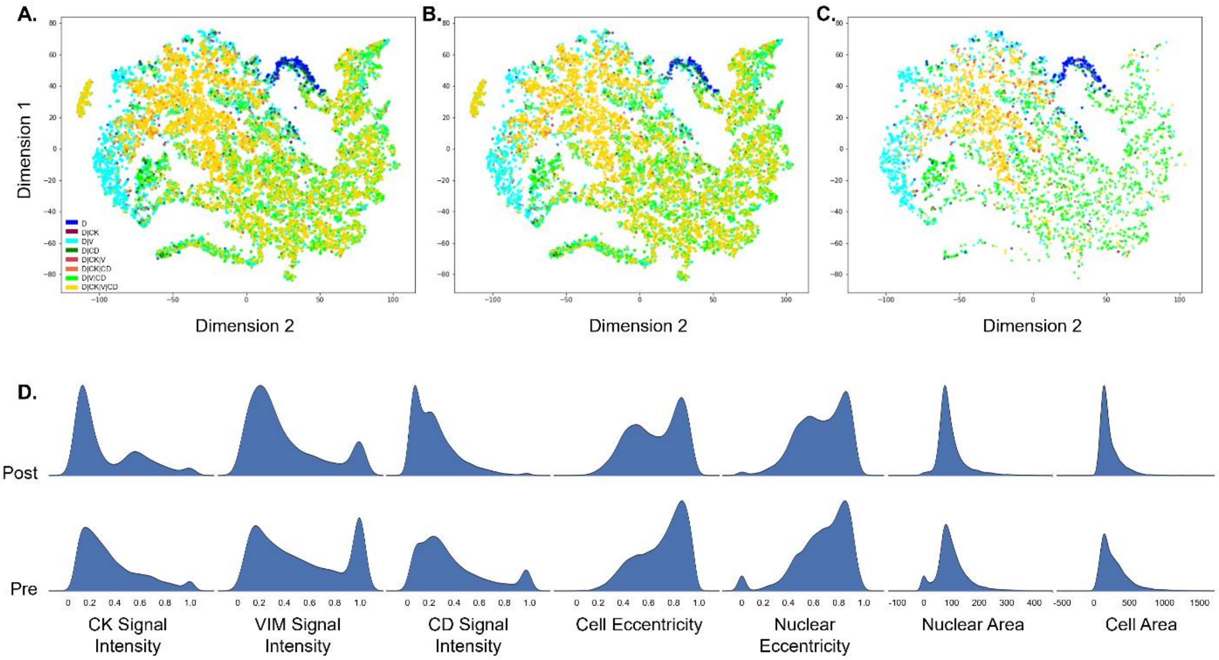

Supplemental Figure 3. Graphical representation of the different channel-type rare cells/mL between timepoints. Morphology tSNE plot of rare cells by channel-type classification in A) and separated by B) Pre-procedure and C) Post-procedure. D) Probability density distribution plots for select morphometric parameters across channel-type classifications of median CK signal intensity, median VIM signal intensity, median CD45/CD31 signal intensity, nuclear eccentricity, nuclear area, cellular eccentricity, and cellular area. The Y-axis remains consistent across all conditions, while X-axis varies depending on the specific parameters. The area under a density curve is always equal to 1. This is because a density curve represents a probability distribution, and the total probability of all possible outcomes within a distribution must sum to 1.

Supplemental Table 1. HDSCA3.0 liquid biopsy rare vent counts/mL for each sample provided as median, mean, range (min and max). D: DAPI, V: VIM, CD: CD45/CD31.

Cohort	Rare event category	median	mean	min	max
**ND**	CK Onc	0.00	0.47	0.00	4.58
	CK|CD Onc	0.00	0.29	0.00	6.55
	CK|V Onc	0.00	0.03	0.00	1.18
	CK|V|CD Onc	0.00	3.34	0.00	123.42
	D	4.00	5.67	0.00	27.19
	D|CD	1.11	2.44	0.00	16.95
	Epi.CTC	0.00	0.21	0.00	2.45
	D|CK|CD	1.20	4.21	0.00	46.60
	Mes.CTC	0.00	0.84	0.00	12.35
	D|CK|V|CD	6.72	14.04	0.00	145.12
	D|V	4.35	8.57	0.00	87.93
	D|V|CD	3.92	11.73	0.00	186.12
	Total Cells	33.57	47.53	4.39	224.52
	Total CK+ Cells	9.96	19.12	0.00	169.56
	Total Events	34.47	51.71	4.39	336.78
	Total Oncosomes	0.32	4.18	0.00	124.53
**PDAC PB**	CK Onc	2.05	5.14	0.00	39.53

	CK|CD Onc	0.00	1.56	0.00	22.79
	CK|V Onc	0.00	0.50	0.00	5.50
	CK|V|CD Onc	0.00	0.26	0.00	3.97
	D	4.06	6.11	0.00	22.78
	D|CD	1.95	3.57	0.00	22.78
	Epi.CTC	0.00	0.94	0.00	13.75
	D|CK|CD	0.00	3.78	0.00	40.41
	Mes.CTC	1.20	4.71	0.00	46.80
	D|CK|V|CD	47.80	202.55	0.00	2192.21
	D|V	27.79	64.64	0.00	493.91
	D|V|CD	41.49	173.54	5.41	2372.95
	Total Cells	192.47	459.84	13.53	5078.39
	Total CK+ Cells	54.62	211.98	0.00	2222.05
	Total Events	208.65	467.39	14.89	5079.22
	Total Oncosomes	3.36	7.55	0.00	39.53
**PDAC PoVB**	CK Onc	33.08	18.74	0.00	139.33
	CK|CD Onc	0.27	0.00	0.00	3.44
	CK|V Onc	0.50	0.00	0.00	5.46
	CK|V|CD Onc	0.22	0.00	0.00	2.73
	D	5.56	3.46	0.00	43.64
	D|CD	1.79	0.00	0.00	28.11
	Epi.CTC	766.52	353.60	0.86	6251.66
	D|CK|CD	91.24	38.62	0.00	479.03
	Mes.CTC	25.54	4.92	0.00	220.49
	D|CK|V|CD	104.13	39.17	0.00	724.04
	D|V	60.93	27.75	1.72	893.44
	D|V|CD	27.31	19.22	1.70	122.06
	Total Cells	1083.03	617.06	37.29	6676.19
	Total CK+ Cells	987.43	560.41	2.71	6554.71
	Total Events	1202.06	674.79	37.29	6768.93
	Total Oncosomes	24.98	119.03	0.00	2396.56

Supplemental Table 2. Significant P-values for the comparison of liquid biopsy analytes between different sample sets. D: DAPI, V: VIM, CD: CD45/CD31.

Comparison	P-value	Event type
PDAC PB vs. PoVB	4.20E-13	Epi.CTC
ND PB vs. PDAC PB	7.57E-12	D|V|CD
ND PB vs. PDAC PB	4.89E-11	D|V
ND PB vs. PDAC PB	4.71E-10	Total Events
ND PB vs. PDAC PB	5.72E-10	Total Cells
PDAC PB vs. PoVB	6.99E-09	D|CK|CD
ND PB vs. PDAC PB	7.04E-08	Total Oncosomes
ND PB vs. PDAC PB	1.17E-07	CK Onc
PDAC PB vs. PoVB	1.04E-06	Total Oncosomes
PDAC PB vs. PoVB	1.15E-06	Total CK+ Cells
PDAC PB vs. PoVB	1.49E-06	CK Onc

ND PB vs. PDAC PB	3.97E-06	D|CK|V|CD
ND PB vs. PDAC PB	1.48E-05	Total CK+ Cells
PDAC PB vs. PoVB	4.16E-05	Mes.CTC
PDAC PB vs. PoVB	0.0001	Total Events
PDAC PB vs. PoVB	0.0001	Total Cells
PDAC PB vs. PoVB	0.0040	D|V|CD
ND PB vs. PDAC PB	0.0070	Mes.CTC
PDAC PB vs. PoVB	0.0204	D|CD
PDAC PB Pre vs Post	0.0495	D|CK|V|CD

Supplemental Table 3. Significant P-values for the comparison of liquid biopsy analytes between different sample sets. Met: metastatic disease, patient samples from EUS cohort. Local: localized disease, patient samples from Surgery and EUS cohort. D: DAPI, V: VIM, CD: CD45/CD31.

Comparison	P-Value	Event Type
**PoVB Met vs. Local**	2.22E-07	Epi.CTC
**PB Met vs. Local**	2.38E-07	D|V|CD
**PoVB Met vs. Local**	5.19E-06	Total Cells
**PoVB Met vs. Local**	5.61E-06	Total CK+ Cells
**PoVB Met vs. Local**	6.55E-06	D|V|CD
**PoVB Met vs. Local**	9.61E-06	Mes.CTC
**PoVB Met vs. Local**	1.12E-05	D|CK|CD
**PoVB Met vs. Local**	1.62E-05	CK Onc
**PoVB Met vs. Local**	2.03E-05	Total Events
**PB Met vs. Local**	0.0035	Total Cells
**PB Met vs. Local**	0.0043	Total Events
**PoVB Met vs. Local**	0.0048	CK|V|CD Onc
**PB Met vs. Local**	0.0180	Mes.CTC
**PB Met vs. Local**	0.0188	Total CK+ Cells
**PB Met vs. Local**	0.0280	D|CK|V|CD
**PoVB Met vs. Local**	0.0331	Total Oncosomes
**PB Met vs. Local**	0.0345	CK Onc
**PB Met vs. Local**	0.0391	D
**PB Met vs. Local**	0.0477	D|V

Supplemental Table 4. Overall survival analysis of rare events in PB and PoVB samples taken during diagnostic workup.

Location	Rare Event	P-Value	Threshold	Threshold Type
**PB**	D|V|CD	0.0010	14.87	first quartile
**PB**	D|V|CD	0.0042	35.56	median
**PoVB**	D	0.0082	3.52	median
**PB**	D|CK|CD	0.0160	4.45	third quartile

**PB**	D|CK|V|CD	0.0217	47.80	median
**PB**	Total CK+ Cells	0.0217	54.62	median
**PB**	Total Cells	0.0419	63.86	first quartile
**PB**	Total Events	0.0419	66.50	first quartile
**PB**	CK|V Onco	0.0446	0.00	first quartile
**PB**	CK|V Onco	0.0446	0.00	median
**PB**	CK|V Onco	0.0446	0.00	third quartile
**PoVB**	D|CD	0.0446	0.00	first quartile
**PoVB**	D|CD	0.0446	0.00	median
